# High Efficient Differentiation of Functional Hepatocytes from Porcine Induced Pluripotent Stem Cells

**DOI:** 10.1371/journal.pone.0100417

**Published:** 2014-06-20

**Authors:** Ying Ao, Jocelyn Danielle Mich-Basso, Bo Lin, Lei Yang

**Affiliations:** 1 Department of Pharmacology, Basic Medical School of Wuhan University, Wuhan, Hubei, China; 2 Department of Developmental Biology, School of Medicine, University of Pittsburgh, Pittsburgh, Pennsylvania, United States of America; University of Tampere, Finland

## Abstract

Hepatocyte transplantation is considered to be a promising therapy for patients with liver diseases. Induced pluripotent stem cells (iPSCs) provide an unlimited source for the generation of functional hepatocytes. In this study, we generated iPSCs from porcine ear fibroblasts (PEFs) by overexpressing Sox2, Klf4, Oct4, and c-Myc (SKOM), and developed a novel strategy for the efficient differentiation of hepatocyte-like cells from porcine iPSCs by following the processes of early liver development. The differentiated cells displayed the phenotypes of hepatocytes, exhibited classic hepatocyte-associated bio-functions, such as LDL uptake, glycogen storage and urea secretion, as well as possessed the metabolic activities of cytochrome P-450 (CYP) 3A and 2C. Furthermore, we compared the hepatocyte differentiation efficacy of our protocol with another published method, and the results demonstrated that our differentiation strategy could significantly improve the generation of morphological and functional hepatocyte-like cells from porcine iPSCs. In conclusion, this study establishes an efficient method for *in vitro* generation of functional hepatocytes from porcine iPSCs, which could represent a promising cell source for preclinical testing of cell-based therapeutics for liver failure and for pharmacological applications.

## Introduction

Liver failure is the final stage of viral hepatitis, hepatic cirrhosis or cancer, causing a high mortality rate in patients. Liver transplantation has been a successful treatment for end-stage liver disease. However, due to the lack of transplantable donors, many patients died on the liver waiting list. Alternatively, hepatocyte transplantation has been proposed to partially recover liver function, and to extend the lifespan of patients until an organ becomes available [1], [2]. Therefore, the availability of an unlimited number of functional hepatocytes could greatly benefit patients with end-stage liver disease. Embryonic stem (ES) cell-derived hepatocytes have been proposed to be a potential cell source for liver regenerative therapy [3], [4]. However, the ethical issues and the potential problem of immune rejection limit the direct application of ES cell-derived hepatocytes in patients. Recently, induced pluripotent stem cells (iPSCs) have been successfully reprogrammed from somatic cells with defined transcriptional factors [5],[6]. iPSCs share the similar characteristics with ES cells and could give rise to all somatic cell types. Therefore, iPSCs-derived hepatocytes could be utilized as a novel and personalized cell source for future liver disease therapy. However, cell replacement therapy for human liver failure has to be pre-clinically tested *in vivo*.

Both rodents and large animals, such as pigs, have been utilized as liver disease models for preclinical and pharmaceutical applications. Among all the available animal models, pigs serve as the most clinically relevant model for human liver disease, given the similarities between pig liver and human liver in morphology, structure and physiology [7], [8], [9]. In 2009, iPS cells were reprogrammed from pig primary ear fibroblasts using four reprogramming genes including Sox2, Klf4, Oct4, and c-Myc [10]. Therefore, the generation of pig iPSCs (piPSCs) and the capability of differentiating piPSCs into hepatocytes, together with the availability of pig liver disease models, have offered a valuable research platform with a wide range of basic and preclinical applications for human liver disease therapy, such as drug toxicity testing, disease modeling and, most importantly, the safety and feasibility testing of autologous transplantation. However, the differentiation of piPSCs to functional hepatocytes has remained poorly studied.

In this study, piPSCs were induced from porcine ear fibroblasts (PEFs) by transferring four reprogramming genes with a lentiviral vector. Additionally, we followed up with the early steps of mammalian liver development to establish a novel differentiation protocol for efficient generation of functional hepatocyte-like cells from piPSCs, which exhibited both morphological and metabolic properties of native pig hepatocytes. 

## Materials and Methods

### Cell culture

All experimental procedures and protocols involving pigs were approved by the Institutional Animal Care and Use Committee of the University of Pittsburgh and conformed to US Animal Welfare Act and institutional guidelines. Briefly, fibroblasts were cultured from porcine ear skin biopsies of an adult male Yucatan Miniature Swine (Sinclair Bio-resources). Small pieces of ear skin tissues were washed in Dulbecco's phosphate-buffered saline (DPBS; Invitrogen) and minced with a surgical blade on a 100 mm Petri dish. Cells were then dissociated from the tissues in 0.25% trypsin-EDTA (Invitrogen) for 10 minutes at 37°C. After three washes, cells were cultured for 6–8 days in Dulbecco's modified Eagle's medium (DMEM; Invitrogen) supplemented with 10% (v/v) fetal bovine serum (FBS, Invitrogen). After removal of unattached clumps of cells by washing the culture plates with DMEM, attached fibroblast cells were further cultured until confluent and passaged using 0.25% trypsin-EDTA for 5 minutes.

### Generation of porcine iPSCs

Cultured porcine fibroblasts with less than 10 passages were seeded at a density of 2×10^5^/cm^2^ in 6-well plates. Fibroblasts were reprogrammed to generate iPS cells by transduction with four human reprogramming factors including Sox2, Klf4, Oct4 and c-Myc within a single lentiviral vector as previously described [11]. Post viral transduction, the cells were grown on gamma irradiation inactivated mouse embryonic fibroblasts (MEFs) feeder layer with porcine iPSCs medium (42.5% DMEM, 30% knock out DMEM [KO-DMEM], 17.5% ES FBS, 10% knock out serum replacement [KSR], 1 mM glutamine, 0.1 mM minimal non-essential amino acids, 50 U and 50 µg/ml penicillin–streptomycin, 50 µM 2-mercaptoethanol, 5 ng/ml basic fetal growth factor [bFGF], 500 U/ml mouse leukemia inhibitory factor [LIF]). Individual colonies emerged at around 3 weeks after transduction and were manually sub-cloned using Pasteur pipettes. Established iPSC clones were passaged every 3–4 days and expanded using porcine iPSC medium. For human or mouse iPSC culture, the cells were grown on gamma irradiation inactivated MEFs feeder layer with human iPSCs medium (DMEM/F12 containing 20% KSR, 2 mM glutamine, 0.1 M nonessential amino acids, 0.1 M 2-mercaptoethanol, 10 ng/ml bFGF, 50 U and 50 µg/ml penicillin-streptomycin) or mouse iPSCs medium (DMEM containing 15% FBS, 2 mM glutamine, 0.1 M nonessential amino acids, 0.1 M 2-mercaptoethanol, 10^3^ U/ml LIF, 50 U and 50 µg/ml Penicillin-Streptomycin). Human or mouse iPSC clones were passaged every 7–8 days or 2–3 days, respectively.

### Alkaline phosphatase (AP) activity

piPSCs were fixed with 4% paraformaldehyde for 15 minutes. After 3 washes, fixed cells were stained with a staining solution containing nitro blue tetrazolium chloride (NBT) and 5-bromo-4-chloro-3-indolyl phosphate toluidine salt (BCIP) (Roche) for 30 minutes at room temperature. The staining was then removed, and cells were rinsed with PBS for 3 times.

### Flow cytometry analysis

piPSCs were harvested and trypsinized by accutase for 2–3 minutes at 37°C. The dissociated single cells were fixed in 4% paraformaldehyde for 10 minutes on ice, washed 3 times with PBS. Cells were blocked with 10% FBS and then incubated with primary antibodies (Cell Signaling): anti-Sox2, anti-Oct4, anti-SSEA4, anti-TRA 1-60 or anti-TRA 1-81, followed with incubation for 1 hour at room temperature with Alex 488-conjugated secondary antibodies (Invitrogen). For SSEA1, blocked cells were incubated with Cy3-conjugated anti-SSEA1 antibody (EMD Millipore) for 1 hour at room temperature. Flow cytometry analysis was carried out with a BD Accuri C6 flow cytometer (Becton Dickinson). Data were analyzed using FlowJo (Treestar).

### Teratoma induction

It was conducted as a service in the Transgenic Core of Magee Women's Hospital, UPMC. Approximately 1×10^6^ piPSCs were suspended in 10 µl Matrigel (BD Biosciences) and injected into the testis of 5-week-old immune-deficient SCID/NOD mice. After 8 weeks, mice were sacrificed, tumors were embedded in paraffin, sectioned and stained with hematoxylin and eosin solutions.

### Karyotyping analysis

Karyotyping was performed by the Cytogenetic Core of the University of Pittsburgh. At least 100 metaphase cells were analyzed, and a minimum of 20 were karyotyped for each line.

### Differentiation of piPSCs to hepatocyte-like cells

Two *in vitro* differentiation protocols were used in this study.

**Figure 1:**
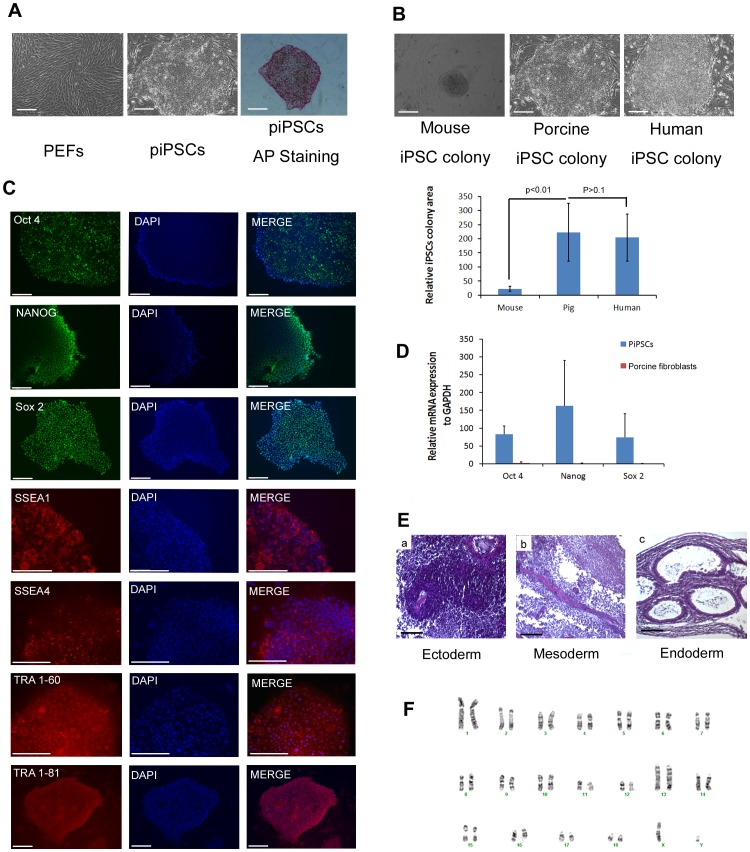
Generation of piPSCs from PEFs. (A) From left to right, morphology of PEFs, an induced piPSCs colony, and an iPSCs colony post AP staining. Scale bars, 200 µm. (B) Typical iPSC colonies from mouse, pig and human before passaging. Scale bars, 200 µm. (C) Expression of pluripotency markers of Oct4, Nanog, Sox2, SSEA1, SSEA4, TRA 1-60, and TRA 1-81 by immunostaining. Scale bars, 200 µm. (D) Q-PCR analysis of pluripotency marker genes Oct4, Nanog and Sox2 in PEFs (red) and piPSCs (blue). The ratio of ΔΔCT was normalized to the internal control GAPDH, error bars represent SEM of three independent experiments. (E) Histological analysis of teratoma derived from piPSCs. Ectoderm (a): pigment epithelium; Mesoderm (b): muscle; Endoderm (c): ciliated columnar epithelium. (F) Karyotyping analysis show the normal karyotype of piPSCs. Error bars show SEM of three independent experiments. P value was calculated using Student's *t*-test.

**Method I:** piPSCs with a 90% confluence were first induced to definitive endoderm (DE) by treating with Roswell Park Memorial Institute (RPMI, Invitrogen) medium containing 100 ng/ml Activin A (PeproTech) and 25 ng/ml Wnt 3a (R&D Systems) for one day (T0), followed by the treatment of cytokine combination of 100 ng/ml Activin A and 10 ng/ml bFGF in serum-free differentiation (SFD) medium for 5 days (T1-T5). To induce hepatoblast formation from DE, the cells were then cultured with SFD medium supplemented with 10 ng/ml bFGF, 50 ng/ml bone morphogenetic protein 4 (BMP4), 10 ng/ml epidermal growth factor (EGF), and 100 ng/ml hepatic growth factor (HGF) (R&D Systems) for 3 days (T6∼T8). During the hepatocyte commitment stage, the cytokines were replaced by 5 µM γ-secretase inhibitor-X, 100 ng/ml HGF, 20 ng/ml oncostatin M (OSM) and 1% dimethyl sulfoxide (DMSO) for 3 days (T9∼T11). Finally, for the maturation of hepatocytes, cells were cultured with SFD containing 100 ng/ml HGF, 20 ng/mL OSM, and 10^−7^ M dexamethasone (Dex) for 6 days (T12∼T18) (Fig. 1A).

**Method II** protocol was adapted from a recent report of human hepatocyte differentiation from human iPS cells [12]. Briefly, when piPSCs had attained a confluence of 70%, the MEF-conditioned medium was replaced by RPMI/B27 with 100 ng/ml Activin A, 50 ng/ml Wnt 3a and 10 ng/ml HGF for 3 days of endodermal induction. During the next step, the culture medium was replaced with hepatic specification medium (knockout [KO]/DMEM containing 20% KSR, 1 mM L-glutamine, 1% nonessential amino acids, 0.1 mM 2-mercaptoethanol, and 1% DMSO). Finally, during the hepatocyte maturation step, the cells were cultured in Iscove's modified Dulbecco's medium (IMDM, Invitrogen) supplemented with 20 ng/ml OSM, 0.5 µM Dex and 50 mg/ml ITS premix (BD Biosciences).

### RNA isolation and quantitative real-time RT-PCR

RNA was extracted using an RNeasy kit (Qiagen) and treated with RNase-free DNase according to manufacturer's instructions. One µg RNA was reverse transcribed to cDNA with high capacity RNA-to-cDNA kit (Applied Biosystems). Quantitative real-time RT-PCR (q-PCR) was performed on an Applied Biosystems 7900HT quantitative PCR system (Applied Biosystems) using Power SYBR Green (Applied Biosystems). Quantification of gene expression was based on the –ΔΔCt method. Glyceraldehyde 3-phosphate dehydrogenase (GAPDH) was used to normalize the messenger RNA levels. Porcine liver tissues were used as the positive control. Primer sequences are listed in Table S1.

### Immunofluorescence

Porcine hepatocyte-like cells were fixed in 4% paraformaldehyde for 20 minutes, and then the cells were permeabilized with 0.1% Triton X-100 for 10 minutes at room temperature. Slides were blocked with 10% normal goat serum (SouthernBiotech) for 30 minutes. The cells were subsequently incubated overnight at 4°C with anti-human albumin (ALB) (Sigma-Aldrich) or anti-human alpha fetoprotein (AFP) (Dako) antibodies at 1∶200 dilution rate in 3% normal goat serum. This was followed by incubation with fluorescein secondary antibody (Invitrogen) for 2 hours at room temperature. Fluorescence images were visualized and captured using a Leica DMRA microscope (Leica).

### Western blot

Cells were trypsinized and collected. Protein was extracted using RIPA buffer (Cell Signaling) with serine protease inhibitor (EMD Millipore) and phenylmethanesulfonylfluoride (PMSF; Cell Signaling). Homogenates were centrifuged, supernatants were collected and stored at −80°C. Proteins were resolved on a 10% Mini-PROTEAN TGX precast SDS-PAGE gel (Bio-Rad) and transferred onto nitrocellulose membranes (Bio-Rad). The membranes were blocked with 10% non-fat dry milk in PBS for 2 hours, followed with overnight incubation at 4°C with the following primary antibodies: anti-human albumin (Sigma-Aldrich), anti-human AFP (Dako), or anti-human GAPDH (Cell Signaling). After 1 hour of incubation with HRP-conjugated secondary antibody (Cell Signaling), the signal was detected using ECL reagents (Thermo Pierce). Porcine liver tissues were used as a positive control.

### Uptake of low-density lipoprotein (LDL)

Cells were incubated for 4 hours with acetylated LDL labeled with 1, 1′-dioctadecyl-3, 3, 3′, 3′-tetramethyl-indocarbocyanine perchlorate (Dil-Ac-LDL; Biomedical Technologies) at 37°C in order to evaluate cellular functionality for LDL uptake. The test was performed according to the manufacturer's instructions after which cells were visualized using a Leica DMRA microscope (Leica).

### Periodic Acid-Schiff (PAS) staining for glycogen

The PAS staining kit was purchased from Sigma-Aldrich. Differentiated cells were fixed in 4% formaldehyde for 20 minutes and then permeabilized with 0.1% Triton X-100 for 10 minutes. The samples were oxidized in 1% periodic acid for 5 minutes, rinsed three times in deionized H_2_O, treated with Schiff's reagent for 30 minutes in dark, and further stained with hematoxylin for 1–2 minutes.

### Urea production

Urea production by the differentiated cells was determined by using the Urea Assay Kit (BioAssay System) according to the manufacturer's instructions. Briefly, 25 µl of culture medium was added into 96-well plates and incubated with 100 µl of Assay Buffer for 50 minutes at room temperature while protected from light. The amount of urea present was measured at OD_430_ using a micro plate reader (Promega). Urea concentration was stated as relative amount/10^5^ cells/ml.

### Cytochrome P450 activities

CYP3A and 2C activities were measured using a P450-Glo Assays kit (Promega) according the manufacturer's instructions. Briefly, the cells were washed with PBS and then treated with fresh medium containing luminogenic CYP3A substrate luciferin-PFBE or CYP2C substrate luciferin-H for overnight incubation at 37°C. To determine CYP P450 enzyme activities, 50 µl of medium was transferred and 50 µl Luciferin Detection Reagent was added to initiate a luminescent reaction for 20 minutes at room temperature. The luminescence of the mixture was then read using a 20/20^n^ luminometer (Torner Biosystem). Cytochrome activity was stated as relative light units (RLU)/10^5^ cells/ml.

### Statistics

All experiments were conducted independently in triplicate. Error bars represent SEM. Statistical significance was estimated with an ANOVA test and *P*-values were calculated using Student's *t*-test. The P values less than 0.05 were considered significant.

## Results

### Generation and characterization of piPSCs

Porcine ear fibroblasts were grown out from an ear skin sample, followed with lentiviral infection of four human reprogramming factors including Sox2, Klf4, Oct4, and c-Myc. Two pig iPS cell clones were established. We observed that it took approximately 3 weeks for porcine iPSC-like clones to emerge post viral transduction, whereas 5–6 weeks was needed for the emergence of human iPSC-like clones from human fibroblast plates. Mouse iPSC-like clones emerged after 2–3 weeks from mouse fibroblast plates post the same lentiviral infection. These indicated a species-specific reprogramming process in mammals. Established piPSCs exhibit a high nucleus-to-cytoplasm ratio with a tightly packed colony formation (Fig. 1A, middle panel), showing a similar morphology to human iPSCs (Fig. 1B, right panel). piPSCs were passaged every 3–4 days, whereas human iPSCs were passaged every 7–8 days and mouse iPSCs were passaged very 2–3 days. Fig. 1B indicates the clone morphology and sizes of porcine, human and mouse iPSC clones right before passaging. We found the size of piPSCs is close to that of human iPSCs, but significantly bigger than that of mouse iPSCs (Fig. 1B).

Pluripotency of piPSCs was validated by AP staining (Fig. 1A, right panel), as well as by immunostaining of nuclear pluripotency markers, such as Oct4, Sox2 and Nanog. Interestingly, we observed that piPSCs expressed the surface markers of both mouse iPSCs, such as SSEA1, and human iPSCs, including SSEA4, TRA 1-60 and TRA 1-81 (Fig. 1C). Flow cytometry analysis also confirmed the expression of these markers in piPSCs (Fig. S1). Q-PCR detected an increased expression level of endogenous porcine pluripotency marker genes including Nanog, Sox2, Oct4 in piPSCs when compared with the primary porcine fibroblasts (Fig. 1D). Additionally, pluripotency was confirmed by the *in vivo* generation of three germ layers from piPSCs using teratoma formation assay (Fig. 1E). Lastly, karyotyping analysis of piPSCs revealed a normal karyotype (Fig. 1F). Taken together, all these data demonstrate the successful generation of pluripotent piPSCs.

### Differentiation of piPSCs into hepatocyte-like cells by following with early liver developmental steps

**Figure 2:**
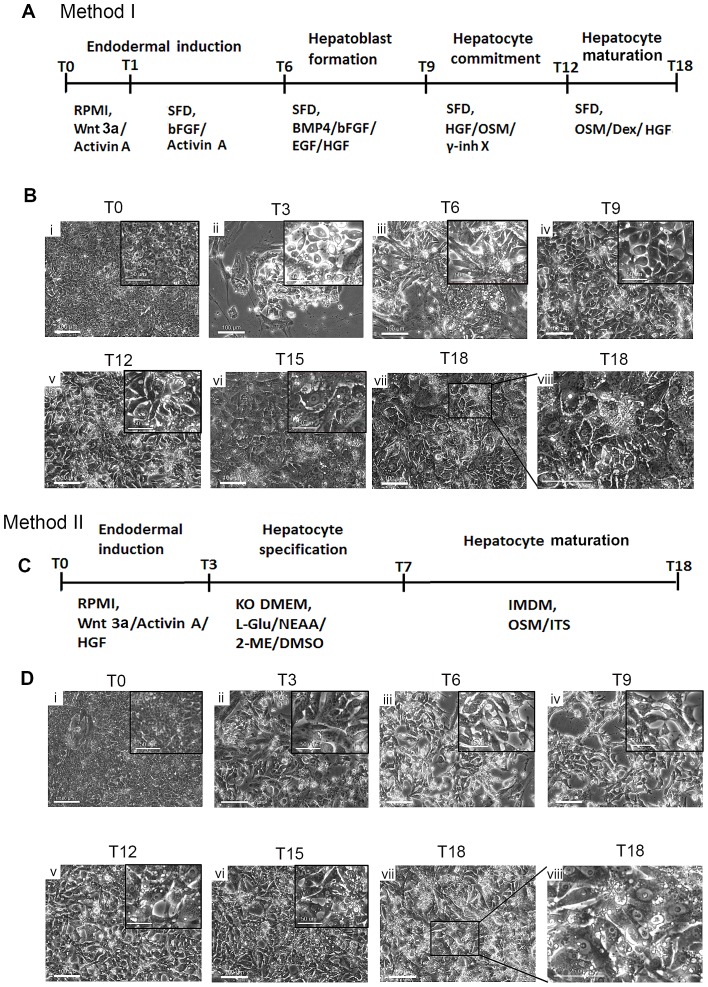
Differentiation of hepatocyte-like cells from piPSCs. (A) A protocol outline describing Method I of hepatocyte differentiation from piPSCs. (B) Morphological changes during piPSCs differentiation to hepatocyte-like cells using Method I. (C) A protocol outline describing Method II of hepatocyte differentiation from piPSCs. (D) Morphological changes during piPSCs differentiation to hepatocyte-like cells using Method II.

In order to efficiently generate hepatocytes from piPSCs, we developed a differentiation protocol (Fig. 2A-Method I) by following the mammalian liver developmental principles. The whole differentiation process was initiated by the induction of endoderm, followed with hepatoblast formation, hepatocyte commitment and the final hepatocyte maturation as previously described [13]. piPSCs were first treated with Activin A, Wnt 3a for 24 hrs (T0-T1), followed with Activin A and bFGF from T1 to T5 to induce definitive endodermal (DE) formation (Fig. 2A). This resulted in the dissociation of cell-cell contact and a change of cell morphology from the compacted iPSCs to a spiky cell shape (Fig. 2B, panel ii and iii). Next, cells were treated with BMP4, bFGF, EGF and HGF from T6 to T8 for hepatic progenitor cell induction. In this period, cells were proliferated, with the morphology changed to spindle or polygonal epithelia-like shape (Fig. 2B, panel iv). Subsequently, cells were induced with the presence of γ-secretase inhibitor-X, HGF and OSM for hepatocyte commitment (T9-T11) and OSM combined with HGF and Dex for maturation (T12-T18). During the final stage, we observed that the cell size increased continuously and changed into a cuboidal shape, with numerous vacuoles and vesicles in the cytoplasm or at the edge of the cells, a large cytoplasmic-to-nuclear ratio, prominent nucleoli, and some cells were found to be binucleated (Fig. 2B, panel v to viii), which are typical morphological features of hepatocytes.

The Method II (Fig. 2C-Method II) was adapted from a previous 3-step protocol for rapid generation of mature hepatocyte-like cells from human iPSCs [12]. In this Method, piPSCs were treated with Activin A, Wnt 3a and HGF from T0 to T3 for inducing endoderm differentiation. We observed the same change of cell morphology as described above in Method I from T0 to T3 (Fig. 2D, panel ii). Next, the cells were cultured with hepatic specification medium for 4 days (T4 to T7). After T8, the cells were treated with OSM, Dex and ITS for hepatocyte commitment and maturation, which resulted in the enlargement of cytoplasmic-to-nuclear ratio, as well as the increased number of small vacuoles in cytoplasm (Fig. 2D, panel v to vii). However, the morphology of T18 cells from Method II remained a spiky or spindle-like shape, which was different from the cuboidal shape of T18 hepatocyte-like cells from Method I (Fig. 2D panel viii vs. Fig. 2B panel viii). Both Method I and II were conducted in 2 piPSC clones and all data shown here were from one representative clone.

### Gene and protein expression profile of the piPSCs-derived hepatocyte-like cells

**Figure 3:**
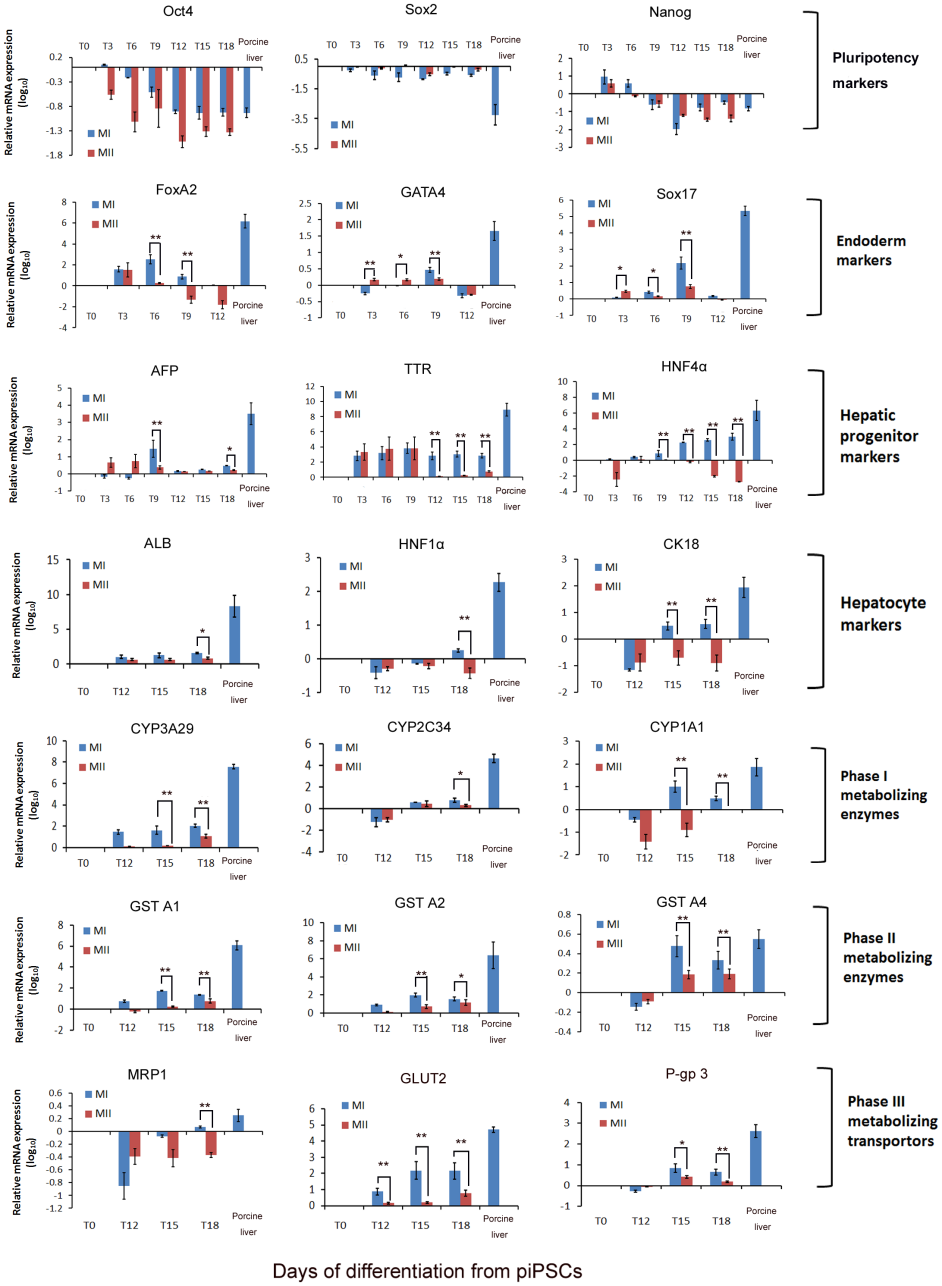
Dynamic gene expressions during hepatic differentiation from piPSCs. Q-PCR analysis of pluripotency marker genes (Oct4, Nanog, and Sox2), definitive endoderm markers (FoxA 2, GATA4 and Sox17), hepatic progenitor markers (AFP, TTR and HNF 4α), hepatocyte markers (ALB, HNF 1α, and CK18), metabolizing phase I enzymes (CYP3A29, CYP2C34, CYP1A1), phase II enzymes (GST A1, GST A2, GST A4) and phase III transporters (MRP1, GLUT2, and P-gp3) at different time points of differentiation with the two hepatic differentiation methods. The ratio of ΔΔCT was normalized to the internal control GAPDH, and fold change results were obtained by normalization to undifferentiated piPSCs on T0. Error bars represent SEM of three independent experiments. ^*^ *P*<0.05, ^**^ *P*<0.01. *t*-test.

To confirm the hepatocyte-induction steps during piPSC differentiation, we examined the relative expression levels of various marker genes using q-PCR (Fig. 3). Porcine liver tissue was used as a positive control, and the fold changes were obtained by normalization to undifferentiated piPSCs at T0. As expected, loss of pluripotency markers of Oct4, Sox2 and Nanog was observed during the differentiation process. In Method I, the expression of endoderm markers, FoxA2, GATA4 and Sox17, significantly increased from T3 to T6, implying the formation of DE from piPSCs. Subsequently, at the hepatoblast formation stage, the expression of hepatic progenitor markers, such as AFP, transthyretin (TTR) and hepatocyte nuclear factor 4α (HNF4α), was markedly increased from T6 to T9. At the hepatocyte commitment (T10–T12) and maturation stages (T12–T18), the expression of hepatic functional marker genes, including ALB, HNF1α, cytokeratin 18 (CK18), transferrin (TFR) and CK8 was increased progressively, whereas the expression of cholangiocyte marker, HNF 1β (Fig. S2), decreased significantly, indicating the specific commitment of hepatic progenitors towards hepatocytes. Moreover, at the final stage (T12-T18), expression levels of metabolizing phase I enzymes (cytochrome P-450 3A29 [CYP3A29], CYP2C34, CYP1A1, CYP2D6), phase II enzymes (such as glutathione S-transferase [GST] A1, GST A2, GST A4, UDP-glucuronosyl-S-transferase [UGT] 1A6) and phase III transporters (namely multidrug-resistant protein 1 [MRP1], glucose transporter 2 [GLUT2] and P-glycoprotein 3 [P-gp3]) were enhanced in the differentiated cells, implying the metabolic potential of piPSC-derived hepatocyte-like cells (Fig. 3 and Fig. S2).

In Method II, the differentiation process was initiated with the endoderm induction stage during the first 3 days (T0 to T3), followed with a hepatic specification stage for 4 more days (T3 to T7) and then a hepatocyte maturation stage from T8 to T18. As shown in Fig. 3, at T3, the expressions of FoxA 2, GATA4 and Sox17 were all markedly induced, which confirmed the DE formation. Subsequently, the expressions of hepatic progenitor markers, AFP and TTR, were significantly increased at T6, suggesting the induction of hepatoblasts. During the final hepatocyte commitment stage (T12–T18), the differentiated cells expressed some of the hepatocyte markers and metabolizing enzymes, such as ALB, TFR, CK8, CYP2A29, CYP2C34, GST A1/A2/A4, GLUT2 and P-gp 3. However, expression levels of those metabolic genes in the end-stage Method II hepatocyte-like cells were significantly lower than those from the Method I cells at the same stage. Thus, these results suggested that although the piPSCs underwent similar liver developmental process when treated with those two differentiation methods, Method I and II led to different efficiencies for hepatic differentiation.

**Figure 4:**
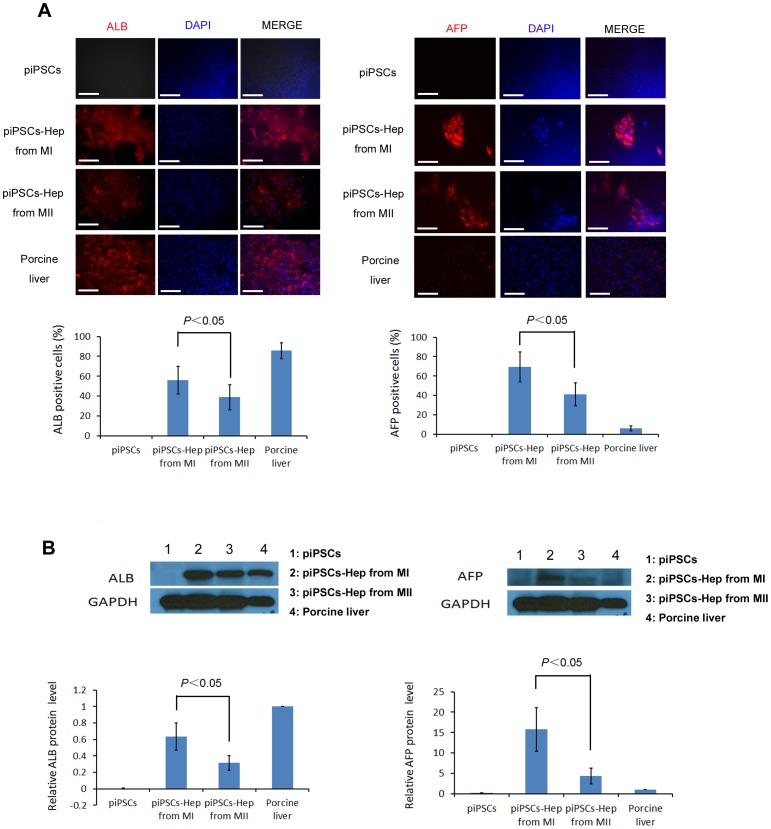
ALB and AFP protein expressions in hepatocyte-like cells. (A) The expressions of ALB and AFP in undifferentiated piPSCs, T18 hepatocyte-like cells generated from Method I & II and pig liver tissue, were detected by immunostaining. Scale bars, 100 µm. The ratios of ALB and AFP positive cells were quantified from approximately 300 cells of each sample. *P*<0.05. (B) The expressions of ALB and AFP on above samples were next examined by using western blot assay. The relative expression ratio was normalized to the internal control GAPDH. Error bars represent SEM of three independent experiments. P value was calculated using Student's *t*-test.

Next, we detected and compared the protein levels of AFP and ALB, which are two markers of hepatocyte differentiation, within the end-stage piPSC-derived hepatocyte-like cells from Method I vs. Method II. Native adult pig liver tissue was used as a positive control. As shown in Fig. 4A upper panel, cytosolic expressions of both ALB and AFP could be detected in the T18 cells differentiated with both Methods. However, the ratio of ALB or AFP positive cells generated from Method I was significantly higher than that from Method II, given that all samples were immunostained and exposed under the same conditions (Fig. 4 lower panel). This result is consistent with the q-PCR data of Fig. 3 and indicates that Method I is more efficient for inducing hepatocyte differentiation than Method II. To further confirm the results of immunostaining, we conducted western blot assay to quantify the protein levels of ALB and AFP in undifferentiated piPSCs, T18 cells from Method I, T18 cells from Method II and adult porcine livers. Significantly increased ALB and AFP levels were found in T18 cells from Method I compared with those of T18 cells from Method II (0.63±0.17 *vs.* 0.31±0.09, *P*<0.05; 15.7±5.3 *vs.* 4.4±2.0, *P*<0.05) (Fig. 4B). Since AFP expression level is high in fetal hepatocytes and decreases during the maturation of fetal liver, the AFP level in adult porcine liver was much lower than those of the T18 cells from both Methods. This result indicates that similar with human iPSC-derived hepatocytes [12], [13], piPSC-derived hepatocyte-like cells are immature.

### Liver-specific bio-functions of piPSCs-derived hepatocyte-like cells

**Figure 5:**
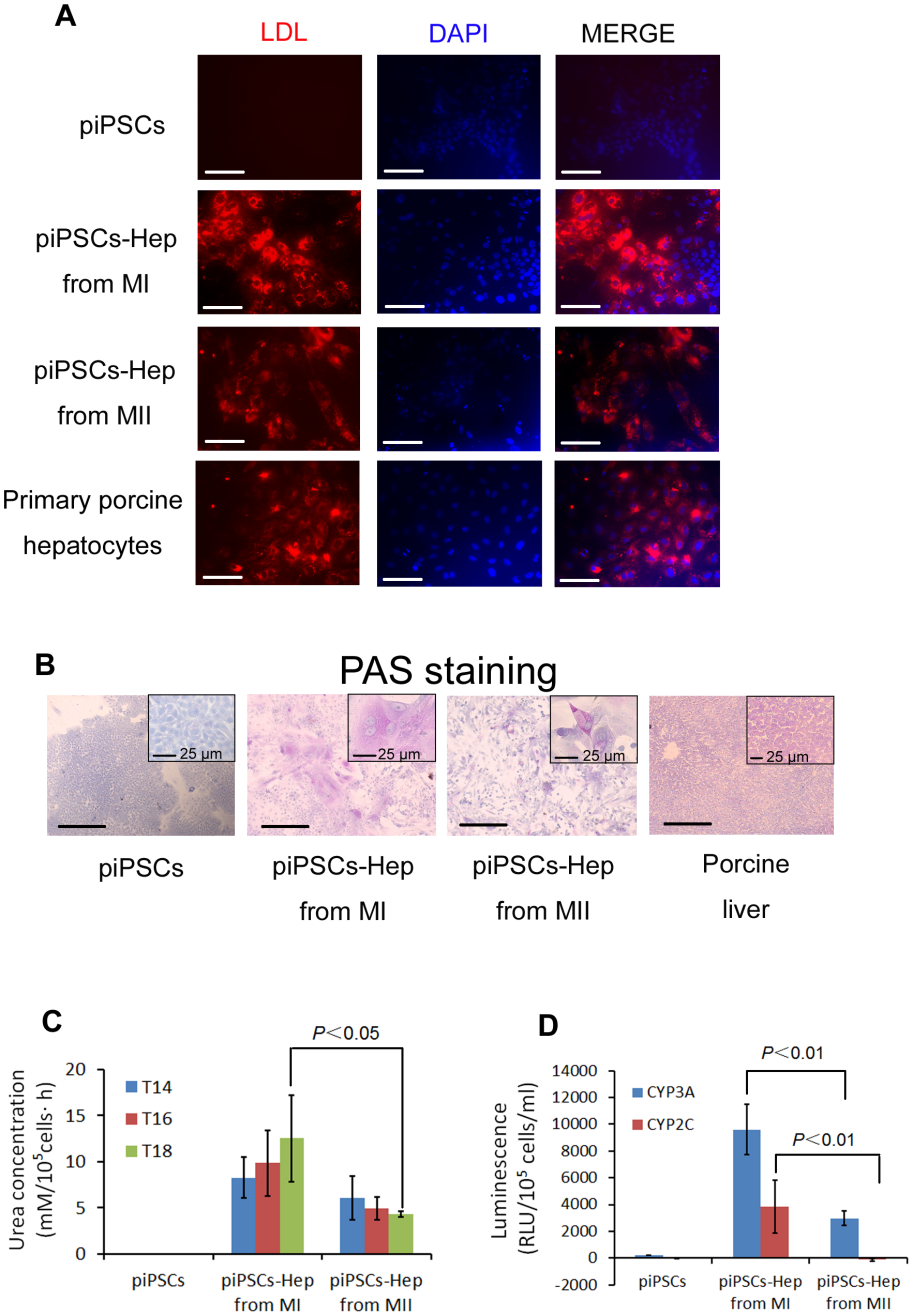
Functional analysis of the hepatocyte-like cells derived from piPSCs. (A) Analyzing LDL uptake on undifferentiated piPSCs, T18 hepatocyte-like cells generated from Method I & II and primary pig hepatocytes. Red fluorescence indicates the cytosolic LDL. Scale bars, 100 µm. (B) PAS staining assay to examine glycogen storage on undifferentiated piPSCs, T18 hepatocyte-like cells generated from Method I & II and pig liver tissue. Scale bars, 200 µm; Scale bars of high magnification images, 25 µm. (C) Urea concentration on T14, T16 and T18 piPSC-derived hepatocyte-like cells using two different differentiation methods. Undifferentiated piPSCs serve as a negative control. (D) Activities of CYP3A and CYP2C on the T18 piPSC-derived hepatocyte-like cells using two differentiation methods. Error bars represent SEM of three independent experiments. P value was calculated using Student's *t*-test. Undifferentiated piPSCs serve as a negative control.

LDL is a lipoprotein that transports cholesterol throughout the body for use by various cells. Most LDL is taken up and metabolized in hepatocytes. Therefore, we first evaluated LDL uptake function of piPSCs-derived hepatocyte-like cells. In Method I cells, the majority of differentiated hepatocyte-like cells were strongly positive for LDL uptake, whereas LDL incorporation was found in much fewer cells generated using method II (Fig. 5A). Next, we examined glycogen storage within piPSCs-derived hepatocyte-like cells, which is an important metabolic function of hepatocytes. By using PAS staining, glycogen was colored magenta in cytoplasm and we found that approximately 80% of piPSC-derived hepatocyte-like cells from Method I were positive for glycogen storage, whereas only 40% of Method II cells and almost none of undifferentiated piPSCs positively responded to PAS staining (Fig. 5B).

Hepatocytes perform numerous detoxification functions, which can be evaluated by testing the ability of cells to synthesis urea, as hepatocytes convert ammonia, a toxic substance, to urea [11]. Therefore, we next assessed the urea concentration from undifferentiated piPSCs, T14, T16 and T18 cells of differentiation. We observed a time-dependent increase of urea concentration in piPSCs-derived hepatocyte-like cells from Method I. However, such an increase was not found in the method II cells. In addition, the urea level at the end stage of differentiation (T18) in Method I cells was significantly higher than that from the Method II cells (Fig. 5C).

Cytochrome P450 enzymes are critically associated with drug metabolism of the liver. CYP3A and 2C are responsible for metabolizing approximately 60% and 19% of drugs in clinic, respectively, and thus are regarded as the most important drug-metabolizing enzymes in hepatocytes. Lastly, we assessed both CYP3A and CYP2C activities in undifferentiated piPSCs and piPSCs-derived T18 hepatocyte-like cells from both Methods. As shown in Fig. 5D, the differentiated cells from Method I showed both CYP3A and CYP2C activities, whereas the Method II cells showed only CYP3A activity and no CYP2C activity. Importantly, a significant increase of CYP3A and CYP2C activities was detected in the hepatocyte-like cells derived from Method I when compared with those from Method II. Taken together, by following the process of early hepatogenesis, we established a robust and efficient differentiation protocol to induce functional hepatocyte-like cells from piPSCs.

## Discussion

The ability of iPSCs to differentiate into most somatic cell types including hepatocytes, represents a promising cellular source for the future treatment of liver failure. Although a variety of hepatic differentiation methods have been established in mouse and human iPSCs [14], [15], [16], less attention has been paid to iPSC-derived from large animals such as pigs. The efficent production of hepacocytes from iPCSs is heavily reliant on the deep understanding of early stage hepatogenesis. In this study, we described a robust hepatocyte differentiation method from porcine iPSCs by following the principles underlying early liver development during mammalian embryogenesis, including DE induction, hepatoblast formation, hepatocyte commitment and maturation [17]. It has been previously reported that Activin A activates Nodal signal for the DE formation in vertebrates [18], [19], and WNT and FGF synergistically interact with Activin A for the DE commitment [20]. In our study, loading of Activin A, Wnt 3a and bFGF during the first 6 days of differentiation significantly enhanced the expressions of Sox17, GATA4 and FoxA 2, indicating the DE induction. BMPs, in parallel to FGF, have been reported as critical growth factors for the growth of hepatic endoderm [21]. Additionally, both HGF and EGF were found to be essential for hepatoblast proliferation [22]. Therefore, adding of BMP4, bFGF, HGF and EGF from differentiation day 6 to day 9 could substantially promote the expansion of early hepatic progenitor cells derived from piPSC-derived DE. GATA4 was reported to remain expressed in the gut endoderm but specifically down-regulated in cells that are specified to a hepatic fate [23]. In this study, we observed a continuously increasing level of GATA4 from day 3 until day 9 of differentiation, followed with a rapid diminishment of GATA4 until day 12 of differentiation, which indicated the hepatic specification from piPSC-derived DE. In early liver formation, hepatoblasts give rise to both hepatocytes and cholangiocytes. Previous studies demonstrated that HGF and γ-secretase inhibitor X could drive the specification of hepatoblasts toward hepatocytes rather than cholangiocytes [24], [25]. Thus the loading of HGF and γ-secretase inhibitor X into piPSC differentiation media from day 9 till day 11 functioned to specify hepatoblasts into hepatocyte fate. OSM has been previously reported to induce hepatocyte maturation by triggering of glucocorticoids [26]. In addition, Dex, a synthetic glucocorticoid, has been found to promote albumin production by suppressing AFP synthesis [27], [28], [29] of the fetal liver. Therefore, adding OSM and Dex into piPSC differentiation media after day 12 until day 18 at hepatocyte maturation stage could enhance the maturation of piPSC-derived hepatocyte-like cells, which was proven by the decrease of AFP but the increase of ALB and other hepatocyte metabolic enzyme genes, as well as the corresponding dynamic morphological change during late-stage hepatic differentiation. All these demonstrated that our strategy facilitated the robust generation of morphological and functional specific hepatocyte-like cells from piPSCs.

Metabolic activity is the most important function of hepatocytes, which is fulfilled through a complex bio-transforming system. In hepatocyte, this system consists of phase I and II metabolizing enzymes and phase III transporters [30]. Our results of Figure 5 demonstrated that piPSC-derived hepatocyte-like cells possessed the metabolic activities of CYP3A and CYP2C, which are the most important enzymes for drug metabolism. Together, these results strongly suggested that the piPSC-derived hepatocyte-like cells with our strategy have the potential for xenobiotics metabolizing function and could be utilized as an *in vitro* model for drug testing.

In this study, we also sought to generate hepatocyte-like cells from piPSCs using a previously published method [12] from human iPSCs. It was reported that this 3-step protocol could rapidly generate mature hepatocytes from human iPSCs with high efficiency. Although this protocol could be utilized to induce hepatic differentiation from piPSCs, end-stage hepatocyte-like cells generated using Method II lacked a typical hepatocyte-like morphology, exhibited low glycogen storage and metabolic activities when compared with hepatocyte-like cells derived from piPSCs using our defined Method I. It is possible that the intrinsic differences between human and porcine iPSCs may result in their different responses to cytokines and subsequently influence the differentiation efficiency, suggesting the need for optimizing species-specific hepatocyte differentiation protocols. In addition, Method I included a dedicated stage for hepatocyte specification (Fig. 2, T9-T12), whereas in Method II this stage was skipped. Lack of this critical stage in Method II may result in the overall decreased expressions of hepatocyte-specific genes and proteins after DE induction, which subsequently led to lower functional activities of end-stage hepatocyte-like cells. Consistent with our result here, a previous study [15] reported a high efficient protocol for making human hepatocyte-like cells from hiPSCs, which included a hepatoctye-specification stage at day 10–15 of hiPSC differentiation. These observations indicated the crucial role of hepatoctye specification during pig and human pluripotent stem cell differentiation.

Although pigs have been serving as a useful large animal model in studying various human diseases, derivation of pig pluripotent stem cell and differentiation of somatic cells from piPSCs have been largely underdeveloped. To date, only one study reported the hepatocyte differentiation from piPSCs [31]. However, the hepatocyte differentiation efficiency is low in that study, and the whole differentiation was not fully characterized in transcriptional and metabolic levels. In addition, the differentiation method of that study was directly adapted from human iPSCs, which is similar to Method II in the current study and lacks species-specific fine-tuning.

Overall, in this study, we generated porcine iPSCs from pig ear fibroblasts and developed an efficient hepatic differentiation strategy for the robust generation of hepatocyte-like cells from piPSCs. The piPSC-derived hepatocyte-like cells in this study possessed the typical phenotypes of pig hepatocytes, as well as liver-specific metabolic functions. The piPSC-derived hepatocyte-like cells could provide an ideal resource to study drug metabolism and have a great potential for preclinical testing of autologous cell transplantation therapy of liver disease using pig liver disease models.

## Supporting Information

Figure S1 **Expression of Sox2, Oct4, TRA 1-81, TRA 1-60 and SSEA1 in piPSCs by flow cytometry analysis.** (A) Fixed cells were incubated with primary antibodies: anti-Sox 2, anti-Oct 4, anti-SSEA4, anti-TRA 1-60 or anti-TRA 1-81, followed with an incubation of 1 hour at room temperature with 488-conjugated secondary antibodies. (B) Fixed cells were incubated with Cy3-conjugated anti-SSEA1 antibody for 1 hour at room temperature.
(TIF)

Figure S2 **Dynamic gene expression patterns of cell markers during differentiation from piPSCs to hepatocyte-like cells.** Q-PCR analysis of cholangiocyte marker HNF 1β, hepatocyte markers (TFR and CK8) and metabolizing enzymes (CYP2D6 and UGT 1A6) during hepatocyte commitment and maturation stages of differentiation by the two methods. The ratio of ΔΔCT was normalized to the internal control GAPDH, and fold change results were obtained by normalization to undifferentiated piPSCs on T0. Error bars represent SEM of three independent experiments. *P<0.05, **P<0.01.
(TIF)

Table S1 **Primer sequences for q-PCR.**
(DOCX)
