# Supplementary figures and images for: High Efficient Differentiation of Functional Hepatocytes from Porcine Induced Pluripotent Stem Cells
Source: PLoS One. 2014 Jun 20;9(6):e100417. doi: 10.1371/journal.pone.0100417 (PMC4065042; doi:10.1371/journal.pone.0100417)

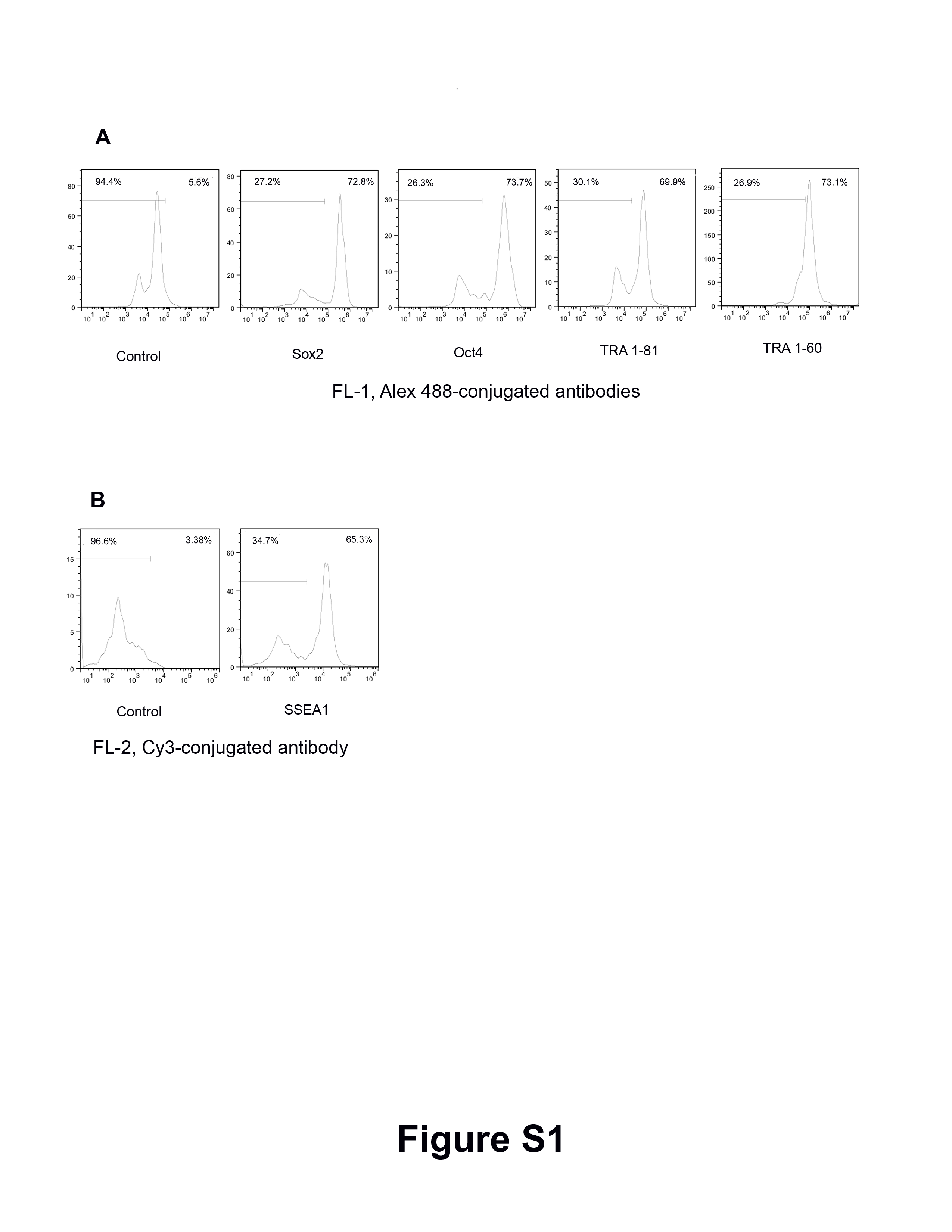

Supplement: Figure S1 — Expression of Sox2, Oct4, TRA 1-81, TRA 1-60 and SSEA1 in piPSCs by flow cytometry analysis. (A) Fixed cells were incubated with primary antibodies: anti-Sox 2, anti-Oct 4, anti-SSEA4, anti-TRA 1-60 or anti-TRA 1-81, followed with an incubation of 1 hour at room temperature with 488-conjugated secondary antibodies. (B) Fixed cells were incubated with Cy3-conjugated anti-SSEA1 antibody for 1 hour at room temperature. (TIF) [file pone.0100417.s001.tif]

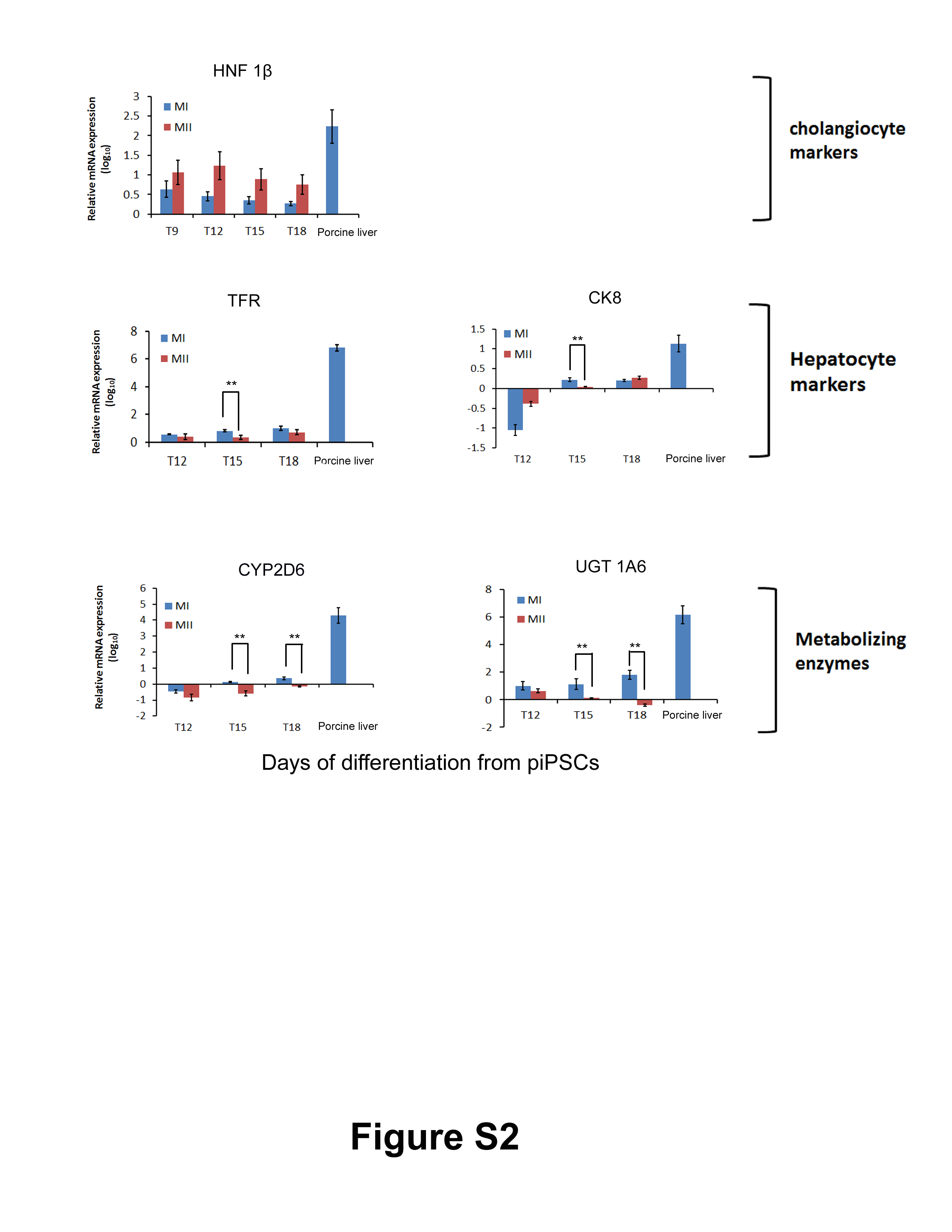

Supplement: Figure S2 — Dynamic gene expression patterns of cell markers during differentiation from piPSCs to hepatocyte-like cells. Q-PCR analysis of cholangiocyte marker HNF 1β, hepatocyte markers (TFR and CK8) and metabolizing enzymes (CYP2D6 and UGT 1A6) during hepatocyte commitment and maturation stages of differentiation by the two methods. The ratio of ΔΔCT was normalized to the internal control GAPDH, and fold change results were obtained by normalization to undifferentiated piPSCs on T0. Error bars represent SEM of three independent experiments. *P<0.05, **P<0.01. (TIF) [file pone.0100417.s002.tif]
