# Supplementary material for: High Efficient Differentiation of Functional Hepatocytes from Porcine Induced Pluripotent Stem Cells
Source: PLoS One. 2014 Jun 20;9(6):e100417. doi: 10.1371/journal.pone.0100417 (PMC4065042; doi:10.1371/journal.pone.0100417)
Supplement: Table S1 — Primer sequences for q-PCR. (DOCX) [file pone.0100417.s003.docx]

**Table S1**

| Gene | Sequence | Product (bp) |
| --- | --- | --- |
| **Oct 4** | F: AGA AGG FCA AAC GAT CAA G  R: AGT ACA GGG TGG TGA AGT | 156 |
| **Sox2** | F: CTG CAG TAC AAC TCC ATG AC  R: GGA GTG GGA AGA AGA GGT AA | 171 |
| **Nanog** | F: GAG CAA CCA AAC CTG GAA  R: GGC CTC CAA ATC ACT GAT AG | 145 |
| **FoxA2** | F: TGC AGA CAC CTC CTA CTA C  R: CCC CTC AAA GTC TCC ATC | 160 |
| **GATA4** | F: CCC AGA CCT TCT CAG TTA GT  R: CCA AGG GTC CTG CTT AGA | 140 |
| **Sox17** | F: GGC CAG AAG CAC AAA CA  R: CAC AAC CTC ACA TTC CTC TC | 162 |
| **AFP** | F: CCC GTC ACG AGT TGT AAA G  R: GCA GCA AGG TGG GAT TAT T | 150 |
| **TTR** | F: GGC TCA CAA CAG ATG AGA AA  R: TGT GGT GGA GTA AGA GTA GG | 188 |
| **HNF4α** | F: GGA GCT GGC AGA AAT GAA  R: CTT GGC ATC TGG GTC AAA | 140 |
| **ALB** | F: CGC TCA TAG TTC GTT ACA CC  R: CCA GGG ACA GAT AGT CTT CA | 150 |
| **HNF1α** | F: CTT CAG AAA GGG AGG GTT TC  R: CGT TTA CCC TCA GCA TCA G | 146 |
| **CK18** | F: TTG ACC GTG GAG TTG GA  R: GAG GTG CTC TCC TCA ATC | 154 |
| **CYP3A29** | F: TTA CAC TTA CCT GCC CTT TG  R: GCC CTT GCG TGG TTA AT | 158 |
| **CYP2C34** | F: CAC CAG ATG GCG TTT ACT T  R: GTC AAT CTC TTT CTG CAC TTT G | 153 |
| **CYP1A1** | F: CAG TAC CAC AAG AGA CAC AAG  R: CCA TCG GCA GTG AGA AAC | 147 |
| **GSTA1** | F: CAG AAG GTG TGG CAG ATT T  R: GTC TTG TCC ATG GCT CTT C | 152 |
| **GSTA2** | F: CAG GGC CAT CCT CAA TTA C  R: GCC ACC TTG GCA TCT TT | 162 |
| **GSTA4** | F: ACG TGA GGA CCG TGT ATA A  R: GAC CAA CTG AGG AAC AAG ATA C | 163 |
| **MRP1** | F: CAT TGT GGC TGT GGA GAG  R: GTA ACG CAA TGC CGT AGT | 144 |
| **Glut2** | F: CAC ATC CTG CTT GGT CTA TC  R: GGT GAC ATC ATC ACT TCC TC | 156 |
| **P-gp3** | F: CCT GTC CAC CAT CCA GAA  R: GAG TTC ATC AGT TCG GTG TC | 149 |
| **HNF1β** | F: CTA CAA CCA GCA GGG AAA C  R: CCA TCA GGT GAG AGG AGA T | 147 |
| **TFR** | F: CTT TCA GGT GTC TGG TTG AG  R: TGG CAG TTC TCA GCA TTA TC | 175 |
| **CK8** | F: GAG TAC CAG GAA CTG ATG AAT G  R: TGG TCT TGG TGT GGA TAC T | 165 |
| **CYP2D6** | F: CTT GGC CAT GGT CAT CTT  R: GTT GAG ACG TGG GTC TTG | 142 |
| **UGT1A6**  **GAPDH** | F: CCC ACT ATC CCA GGA ATT TG  R: CAG AGG ACT GTC TGA GGT AT  F: GAA CAT CAT CCC TGC TTC TAC  R: CTG CTT CAC CAC CTT CTT G | 153  181 |
